# P02.169. The effects of massage therapy on Multiple Sclerosis patients

**DOI:** 10.1186/1472-6882-12-S1-P225

**Published:** 2012-06-12

**Authors:** B Schroeder, K Premkumar, J Doig

**Affiliations:** 1 University of Saskatchewan, Saskatoon, Canada

## Purpose

Mobility difficulties are prevalent in patients with Multiple Sclerosis (MS) and often result in a loss of independence and a diminished quality of life. Massage therapy is a non-invasive supplemental treatment that many MS patients utilize to assist in their symptom management. We explored the effect of massage therapy on mobility and overall quality of life (QoL) of MS patients.

## Methods

Twenty-four MS patients with scores ranging from 3.0 to 7.0 on the Expanded Disability Status Scale (EDSS) received four weeks of Swedish massage treatments. The Six-Minute-Walk-Test (6MWT) was used to assess their exercise capacity and leg function and the Hamburg Quality of Life in MS (HAQUAMS) instrument was used to assess changes in client QoL. These assessments were measured before and after a massage period and a rest period where no massages were employed.

## Results

The results displayed no significant changes in 6MWT distances or HAQUAMS scores after massage or rest periods. However, clients’ personal health rating improved after massage and deteriorated when massages were removed. Client comments collected at the end of the study supported this change. The improvement in patient perception could have been due to an analgesic effect of massage that decreases pain. In addition, the relaxation induced by massage is very beneficial in stress management and thus symptom management for MS individuals.

## Conclusion

Although the results from this study display a limited significant change after massage treatments, it is important to note that no harm was being done. Thus, massage is a safe, non-invasive supplementary treatment option that may assist MS patients to manage the stress of their symptoms and improve quality of life.

